# Effect of ApoE ε4 gene polymorphism on the correlation between serum uric acid and left ventricular hypertrophy remodeling in patients with coronary heart disease

**DOI:** 10.3389/fcvm.2022.1055790

**Published:** 2022-12-21

**Authors:** Jia Liu, Mei-Li Zheng, Mulei Chen, Kuibao Li, Xiaoming Zhu, Yuanfeng Gao

**Affiliations:** Heart Center and Beijing Key Laboratory of Hypertension, Beijing Chaoyang Hospital, Capital Medical University, Beijing, China

**Keywords:** apolipoprotein E, polymorphism, uric acid, left ventricular hypertrophy, heart failure

## Abstract

**Background:**

Hyperuricemia and dyslipidemia are associated with left ventricular hypertrophy (LVH), while the effect of ApoE gene polymorphism on the correlation between serum uric acid (UA) level and severity of LVH in patients with coronary heart disease (CHD) has not been clarified.

**Methods:**

This was a retrospective observational study of patients with CHD. Patients were divided into groups of ε4 carriers and non-ε4 carriers based on sanger sequencing. The association of ApoE ε4 gene polymorphism, serum UA level, and LVH, determined by cardiac color Doppler ultrasound, was evaluated by multivariate analysis.

**Results:**

A total of 989 CHD patients who underwent ApoE genotyping were enrolled and analyzed. Among them, the frequency of the ApoE ε4 genotype was 17.9% (15.7% for E3/4, 1.1% for E4/4, and 1.1% for E2/4). There were 159 patients with LVH, 262 with end-diastolic LV internal diameter (LVEDD) enlargement, 160 with left ventricular ejection fraction (LVEF) reduction, and 154 with heart failure. Multivariate analysis showed that for every increase of 10 μmol/L in serum UA level, the risk of LVH decreased in ε4 carriers (odds ratio (OR) = 0.94, 95% confidence interval (CI): 0.890–0.992, *P* = 0.025) and increased in non-ε4 carriers (OR = 1.03, 95% CI: 1.005–1.049, *P* = 0.016). The risk of LVEDD enlargement tended to decrease in ε4 carriers (OR = 0.98, 95% CI: 0.943-1.023, *P* = 0.391) and increased in non-ε4 carriers (OR = 1.03, 95% CI: 1.009–1.048, *P* = 0.003). The risk of LVEF reduction was reduced in ε4 carriers (OR = 0.996, 95% CI: 0.949–1.046, *P* = 0.872) and increased in non-ε4 carriers (OR = 1.02, 95% CI: 0.994–1.037, *P* = 0.17). The risk of LVEDD enlargement decreased in ε4 carriers (OR = 0.98, 95% CI: 0.931–1.036, *P* = 0.508) and increased in non-ε4 carriers (OR = 1.02, 95% CI: 0.998–1.042, *P* = 0.07).

**Conclusion:**

High serum UA levels decreased the risk of LVH in ApoE ε4 carriers with CHD, while increased the risk of LVH in non-ε4 carriers.

## Introduction

Heart failure (HF) is one of the leading causes of hospitalization and death worldwide, and progressive left ventricular remodeling plays an important role in the pathophysiology of HF. Left ventricular remodeling is characterized by left ventricular hypertrophy (LVH), atrioventricular dilation, interstitial fibrosis, and worsening of cardiac dysfunction (1, 2). LVH, as one of the important phenotypes of left ventricular remodeling, is not only the main adverse myocardial reaction for long-term overloaded pressure load (3), but also is an early clinical marker of cardiovascular disease (CVD), and it is an important predictor of cardiovascular morbidity and mortality evaluated by echocardiography (4). Meanwhile, LVH is also one of the independent risk factors, influencing the prognosis of coronary heart disease (CHD). A series of symptoms, such as myocardial ischemia caused by coronary artery stenosis, elevated pulse pressure, and reduced cardiac diastolic function, cause hemodynamic abnormalities in patients with coronary disease. Long-term effects can lead to the increase of left ventricular pressure and volume, LVH, and left ventricular enlargement, which can progress to the left ventricular dysfunction and HF, resulting in the poor prognosis and decreased quality of life, and increase the economic burden on the healthcare systems. The aim of LVH management is to control the etiology of LVH (5, 6). Therefore, early detection of LVH and control of risk factors are of great significance to both patients and society.

LVH is not only mediated by the mechanical stress of pressure overload, but also by various neurohormonal factors (7), including neuro-humoral factors, and involvement of paracrine/autocrine cardiovascular factors and insulin resistance, which may stimulate the production of cytokines and growth factors that induce cardiac protein synthesis and hypertrophy. Hyperuricemia and lipid abnormalities are both important risk factors for LVH through metabolic abnormalities (8, 9), and serum uric acid (UA) levels can be used as a predictor of echocardiographic parameters in the long-term process from a normal left ventricular mass index (LVMI) to LVH (10, 11), while the underlying mechanism has not been completely clarified. Previous studies have shown that apolipoprotein E (ApoE) gene polymorphism plays a role in the development of gout, primary hyperuricemia, and hypertriglyceridemia. The most widely-studied gene variant in relation to CVD is the apoE e(ε2, ε3, ε4) polymorphism. The three alleles would result in 6 genotypes, namely e3/e3, e3/e4, e2/e3, e2/e4, e4/e4, and 2/e2. While ε2 and ε4 have opposite effects on APOE concentrations, so it was excluded from the APOE allele grouping in the main analysis (12). In particular, the ε4 allele can significantly increase the concentration of total cholesterol in healthy individuals (13, 14), thus, it may cause atherosclerosis, and accelerate progression of CHD to HF (15). ApoE gene polymorphism is associated with the risk of hyperuricemia in various populations (16, 17). With the continuous in-depth studies on ApoE gene polymorphism and the popularization of clinical investigations, it was found that patients with different ApoE gene polymorphisms have different left ventricular remodeling progressions to hyperuricemia. However, the effect of ApoE gene polymorphism on the correlation between serum UA level and LVH has still remained elusive. Therefore, the present study aimed to: (1) investigate the association between serum UA level and LVH; (2) examine the effect of ApoE polymorphism on the association between serum UA level and LVH in CHD.

## Methods

### Study population

This retrospective observational study included patients with CHD who were admitted to the Beijing Chaoyang Hospital (Beijing, China) between January 2015 and December 2016. The inclusion criteria were as follows: (1) patients who aged >18 years old; (2) patients who were angiographically diagnosed with CHD; (3) patients who underwent echocardiography; (4) patients with available ApoE polymorphism status. The exclusion criteria were as follows: (1) severe organ failure and malignant diseases; (2) patients with the ε4/2 genotype [in previous studies, patients with e4/2 genotype were excluded from subsequent analyses because the ε2/4 alleles are proposed to have opposite effects on CHD risk (12)]; or (3) patients with missing covariates. Patients were grouped according to their ApoE genotype: ε4 carriers, and non-ε4 carriers. The present study was approved by the Clinical Research Ethic Committee of Beijing Chaoyang Hospital, the ethics approval number is 2020 - KE - 509.

### Laboratory tests

Genomic DNA extracted from whole-blood specimens was analyzed for ApoE polymorphisms using polymerase chain reaction-reverse hybridization (18). Plasma ApoE levels were measured by nephelometry. Serum UA, creatinine, total cholesterol (TC), triglycerides (TG), low-density lipoprotein cholesterol (LDL-C), N-terminal pro-B-type natriuretic peptide (NT-BNP), glucose, and hemoglobin A1c (HbA1c) levels were determined by standard clinical techniques, following 9-12-ing plasma glucose (FPG) ≥7 mmol/L, HbA1c ≥6.5%, and/or taking oral hypoglycemic agents or receiving insulin injection that were considered to be diabetic (19). Subjects with dyslipidemia were those with LDL ≥2.6 mmol/L, TG ≥1.7 mmol/L, HDL <1 mmol/L, and/or those taking lipid-lowering drugs (20, 21).

### Outcomes

LVH was defined as LVMI ≥125 g/m^2^ (males) or LVMI ≥110 g/m^2^ (females) using the 2007 Guidelines for the Management of Arterial Hypertension presented by the European Society of Hypertension (ESH) and of the European Society of Cardiology (22). LVEDD enlargement was defined as LVEDD ≥50 mm (the 75th percentile of LVEDD, which was calculated from 989 participants). Left ventricular ejection fraction (LVEF) reduction was defined as LVEF <60% (the 20th percentile of LVEDD, which was calculated from 989 participants). Diagnosis of heart failure was based on criteria according to the 2016 ESC heart failure guideline. Briefingly, patients with reduced LVEF (<50%) would be diagnosed with HF if she or he presented with typical symptoms (like orthopnea) or chest radiograph signs of pulmonary edema, or elevated BNP. In the present study, 160 patients were found to have reduced LVEF, while six of them did not meet the HF diagnosis criteria without other diagnosis signs (23).

### Statistical analysis

Normally distributed continuous variables were expressed as the mean ± standard deviation (SD), and were analyzed using the Student's *t*-test (normality was tested using the Kolmogorov-Smirnov test); abnormally distributed, continuous variables were expressed as median (interquartile range) or tertile, and were analyzed using a non-parametric test (Wilcoxon test or Mann-Whitney U test, as appropriate). Categorical variables were expressed as percentage and number, and were analyzed using the Chi-square test. Logistic regression analysis of associations of serum UA level with LVH, LVEDD enlargement, LVEF reduction, and HF was performed using univariable and multivariable models. The interaction terms between serum UA level and ApoE genotype groups for all outcomes were evaluated. The statistical analysis was performed using SPSS 21.0 software (IBM, Armonk, NY, USA). A two-sided *P* < 0.05 was considered statistically significant.

## Results

### Patients' characteristics

**Figure 1:**
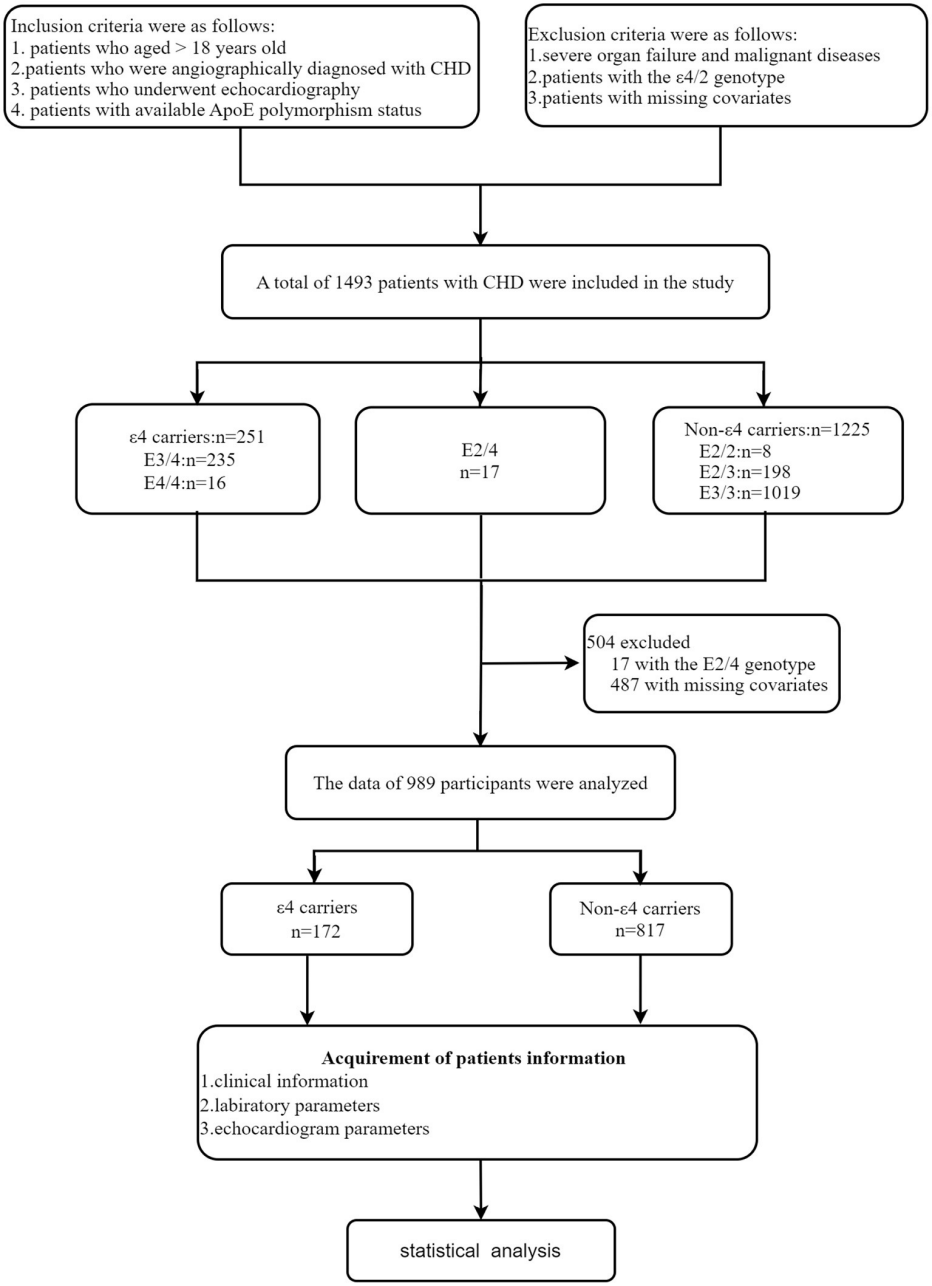
Study design of the present work.

A total of 1,493 patients with CHD were included in the study, of whom 17 patients with the E4/2 genotype were excluded, and 487 patients with missing covariates were ruled out. The data of 989 participants were analyzed (Figure 1). Among all patients, the frequencies of the ApoE genotypes were 15.7% for E3/4, 1.1% for E4/4, 1.1% for E2/4, 0.5% for E2/2, 13.3% for E2/3, and 68.3% for E3/3. There were no significant genotype-based differences between males and females. The subgroups were divided by ApoE genotypes, ε4 carriers, and non-ε4 carriers, respectively.

Participants' demographic and biochemical characteristics are summarized in Table 1. In males, no significant differences were found between the ε4 carriers and the non-ε4 carriers. In females, compared with the non-ε4 carriers, the ε4 carriers were younger (63.8 ± 10.4 vs. 67.1 ± 11.9 years old, *P* = 0.043) and had a higher LDL-C level (2.8 ± 1.1 vs. 2.5 ± 0.8 mmol/L, *P* = 0.032), a lower HbA1c level (6.3 ± 3.3% vs. 6.8 ± 1.7%, *P* = 0.047), a higher maximum diastolic blood pressure (DBP) (103 ± 20 vs. 95 ± 14 mmHg, *P* = 0.006), and a lower current systolic blood pressure (SBP) (129 ± 19 vs. 136 ± 19 mmHg, *P* = 0.009).

**Table 1:** Characteristics of the study population.

	**Male (*****n*** = **643)**			**Female (*****n*** = **346)**		
	**ε4 carriers (*N* = 109)**	**Non-ε4 carriers (*N* = 534)**	***P***	**ε4 carriers (*N* = 63)**	**Non-ε4 carriers (*N* = 283)**	***P***
Age, years	61.3 ± 12.0	61.5 ± 12.5	0.839	63.8 ± 10.4	67.1 ± 11.9	0.043
Heart rate, beats/min	74.5 ± 16.0	74.4 ± 13.9	0.952	75.0 ± 13.1	73.8 ± 13.2	0.506
UA, μmol/L	364.2 ± 113.9	365.9 ± 101.5	0.877	300.8 ± 82.5	317.0 ± 91.7	0.199
Creatinine, μmol/L	67.6 (58.8–79.2)	71.4 (62.9–83.2)	0.046	56.4 (50.5–65.5)	55.9 (47.9–66.8)	0.557
eGFR, ml/min/1.73 m^2^	109 ± 32	104 ± 32	0.18	101 ± 38	102 ± 36	0.89
TC, mmol/L	4.3 ± 1.1	4.2 ± 1.1	0.193	4.6 ± 1.1	4.4 ± 1.0	0.134
TG, mmol/L	1.8 ± 1.4	1.8 ± 1.5	0.989	1.6 ± 0.9	1.7 ± 1.1	0.637
LDL-C, mmol/L	2.6 ± 0.8	2.4 ± 0.9	0.176	2.8 ± 1.1	2.5 ± 0.8	0.032
Dyslipidemia, *n* (%)	67 (61.5)	363 (68.0)	0.188	41 (65.1)	189 (66.8)	0.795
Statins, *n* (%)	12 (11.0)	71 (13.3)	0.516	8 (12.7)	43 (15.2)	0.613
NT-BNP, pg/ml	121.7 (47.2–673.4)	133.9 (48.9–695.5)	0.996	131.9 (59.2–474.0)	146.6 (64.7–394.4)	0.758
Glucose, mmol/L	6.3 ± 2.6	7.0 ± 3.4	0.061	6.6 ± 3.3	6.8 ± 4.0	0.727
HbA1C, %	6.7 ± 1.8	6.7 ± 1.8	0.998	6.3 ± 1.2	6.8 ± 1.7	0.047
Diabetes, *n* (%)	40 (36.7)	204 (38.2)	0.768	25 (39.7)	136 (48.1)	0.228
Highest SBP, mmHg	173 ± 24	174 ± 24	0.748	183 ± 27	179 ± 22	0.293
Maximum DBP, mmHg	102 ± 17	101 ± 17	0.75	103 ± 20	95 ± 14	0.006
Current SBP, mmHg	134 ± 16	133 ± 20	0.828	129 ± 19	136 ± 19	0.009
Current DBP, mmHg	77 ± 12	76 ± 12	0.698	72 ± 11	73 ± 11	0.36
Hypertension, *n* (%)	72 (66.1)	353 (66.1)	0.992	44 (69.8)	199 (70.3)	0.94
ACEI/ARB, *n* (%)	23 (21.1)	142 (26.6)	0.232	19 (30.2)	88 (31.1)	0.884
BB, *n* (%)	13 (11.9)	59 (11.0)	0.791	11 (17.5)	45 (15.9)	0.761
Smoking status, *n* (%)			0.164			0.49
Current	44 (40.4)	262 (49.1)		2 (3.2)	14 (4.9)	
Ever	36 (33.0)	134 (25.1)		1 (1.6)	12 (4.2)	
Never	29 (26.6)	138 (25.8)		60 (95.2)	257 (90.8)	

Normally distributed continuous variables were expressed as the mean ± SD, and abnormally distributed continuous variables were expressed as median (interquartile range); categorical variables were expressed as number and percentage. UA, uric acid; eGFR, estimated glomerular filtration rate; TC, total cholesterol; TG, triglycerides; LDL-C, low-density lipoprotein cholesterol; NT-BNP, N-terminal pro–B-type natriuretic peptide; HbA1C, hemoglobin A1c; SBP, systolic blood pressure; DBP, diastolic blood pressure; ACEIs/ARBs, angiotensin converting enzyme inhibitors/angiotensin-receptor blockers; BB, beta-blockers.

### Cardiac indicators

The comparison of outcomes grouped by gender and ApoE genotype is shown in Table 2. In both genders, there were no significant differences in LVMI, LVEDD, LVEF values, frequencies of LVH, LVEDD enlargement, LVEF reduction, and HF between the ε4 carriers and non-ε4 carriers.

**Table 2:** Comparison of cardiac indicators.

	**Male (*****n*** = **643)**			**Female (*****n*** = **346)**		
	**ε4 carriers (*N* = 109)**	**Non-ε4 carriers *N* = 534)**	***P***	**ε4 carriers (*N* = 63)**	**Non-ε4 carriers (*N* = 283)**	**iP**
**LVMI, g/m** ^**2**^						
Mean ± SD	104.1 ± 30.9	101.1 ± 27.3	0.314	92.1 ± 25.1	93.9 ± 27.9	0.638
Median (25th−75th)	98.7 (84.3–116.3)	95.8 (82.6–114.1)		90.8 (76.3–104.8)	89.5 (76.1–106.3)	
LVH, *n* (%)	19 (17.4)	76 (14.2)	0.391	11 (17.5)	53 (18.7)	0.815
**LVEDD, mm**						
Mean ± SD	49.1 ± 5.3	48.4 ± 5.6	0.231	45.3 ± 6.6	45.6 ± 4.4	0.633
Median (25th−75th)	48.0 (46.0–51.0)	48.0 (45.0–51.0)		45.0 (43.0–48.0)	46.0 (43.0–48.0)	
LVEDD enlargement, *n* (%)	43 (39.4)	190 (35.6)	0.444	7 (11.1)	22 (7.8)	0.387
**LVEF, %**						
Mean ± SD	64.5 ± 10.6	64.2 ± 10.4	0.808	67.3 ± 8.1	67.0 ± 7.5	0.817
Median (25th−75th)	67.0 (60.0–72.0)	67.0 (61.0–71.0)		68.0 (63.0–73.0)	68.0 (64.0–72.0)	
LVEF reduction, *n* (%)	22 (20.2)	113 (21.2)	0.819	5 (7.9)	20 (7.1)	0.789
HF, *n* (%)	17 (15.6)	101 (18.9)	0.415	6 (9.5)	30 (10.6)	0.8

LVMI, left ventricular mass index; LVH, left ventricular hypertrophy; LVEDD, end-diastolic left ventricular internal diameter; LVEF, left ventricular ejection fraction; HF, heart failure.

### Serum UA level and LVH

There were 159 patients with LVH and 262 patients with LVEDD enlargement among the 989 patients. In both univariable and multivariable models, a high serum UA level increased the risk of LVH and LVEDD enlargement (Table 3). After adjusting for gender, age, heart rate, hypertension, diabetes, dyslipidemia, estimated glomerular filtration rate (eGFR), history of receiving angiotensin converting enzyme inhibitors (ACEIs)/angiotensin receptor blockers (ARBs)/β-blockers/statins, and smoking status, the risk of LVH and LVEDD enlargement was escalated by 2 and 4%, respectively, for every increase of 10 μmol/L in serum UA level (Table 3).

**Table 3:** Univariate and multivariate regression analyses of the effect of serum UA level on left ventricular remodeling and cardiac function.

**Dependent variables**	**UA as independent variable (Every 10 μmol/L)**	**Events/Total**	**OR**	**95% CI**	***P*-value**	***P* for interaction**
**LVH**						
	Univariable	159/989	1.02	1.005–1.038	0.009	
	Multivariable^*^	159/989	1.02	1.004–1.039	0.015	
	ε4 carriers group^*^	30/172	0.94	0.890–0.992	0.025	0.013
	Non-ε4 carriers group^*^	129/817	1.03	1.005–1.049	0.016	
**LVEDD enlargement**						
	Univariable	262/989	1.04	1.027–1.056	<0.001	
	Multivariable^*^	262/989	1.03	1.012–1.043	<0.001	
	ε4 carriers group^*^	50/172	0.98	0.943–1.023	0.391	0.415
	Non-ε4 carriers group^*^	212/817	1.03	1.009–1.048	0.003	
**LVEF reduction**						
	Univariable	160/989	1.03	1.014–1.047	<0.001	
	Multivariable^*^	160/989	1.02	1.002–1.037	0.032	
	ε4 carriers group^*^	27/172	0.996	0.949–1.046	0.872	0.602
	Non-ε4 carriers group^*^	133/817	1.02	0.994–1.037	0.17	
**HF**						
	Univariable	154/989	1.03	1.012–1.045	0.001	
	Multivariable^*^	154/989	1.02	1.007–1.042	0.007	
	ε4 carriers group^*^	23/172	0.98	0.931–1.036	0.508	0.134
	Non-ε4 carriers group^*^	131/817	1.02	0.998–1.042	0.07	

* Models adjusted for age, gender, heart rate, dyslipidemia, hypertension, diabetes, eGFR, history of receiving ACEIs/ARBs/BB/statins, and smoking status. LVH, left ventricular hypertrophy; LVEDD, end-diastolic left ventricular internal diameter; LVEF, left ventricular ejection fraction; HF, heart failure; UA, uric acid.

Multivariate analysis of ApoE genotype showed that after adjusting for age, gender, heart rate, dyslipidemia, hypertension, diabetes, eGFR, history of receiving ACEIs/ARBs/β-blockers/statins, and smoking status, the risk of LVH decreased in the ε4 carriers [odds ratio (OR) = 0.94, 95% confidence interval (CI): 0.890–0.992, *P* = 0.025] and increased in the non-ε4 carriers (OR = 1.03, 95% CI: 1.005–1.049, *P* = 0.016) as serum UA level increased (for every increase of 10 μmol/L). The interaction effect for LVH between serum UA level and ApoE genotype groups was significant (*P* (for interaction) = 0.013). The risk of LVEDD enlargement tended to decrease in the ε4 carriers (OR = 0.98, 95% CI: 0.943–1.023, *P* = 0.391) and significantly increased in the non-ε4 carriers (OR = 1.03, 95% CI: 1.009–1.048, *P* = 0.003) (Table 3).

### Serum UA level and cardiac function

There were 160 patients with LVEF reduction and 154 patients with HF among the 989 patients. In both univariable and multivariable models, a higher serum UA level increased the risk of LVEF reduction and HF. After adjusting for gender, age, heart rate, hypertension, diabetes, dyslipidemia, eGFR, history of receiving ACEIs/ARBs/β-blockers/statins, and smoking status, the risk of LVEF reduction and HF was escalated by 2 and 2%, respectively, for every increase of 10 μmol/L in serum UA level (Table 3).

## Discussion

Hyperuricemia, hypercholesterolemia, and ApoE gene polymorphism are all important risk factors, affecting left ventricular remodeling. Data analysis in our study showed that hyperuricemia increased the risk of LVH, LVEDD enlargement, LVEF reduction, and HF in patients with CHD, which is consistent with results of a previous research (9). Serum UA level may be the mechanism of LVH promotion in UA activation to produce a large number of oxygen free radicals within the cell, hydrogen peroxide, such as reactive oxygen species (ROS), which leads to endothelial injury, increases the oxidative stress, and promotes the oxidized low-density lipoprotein and lipid peroxidation. High levels of serum UA can be associated with the activation of renin-angiotensin system, promoting the angiotensin II. It can also inhibit the release of nitric oxide from vascular endothelium (24) and promote the increase of endothelin in blood circulation (25), resulting in changes in vascular endothelial function and the increase of peripheral resistance to vascular contraction. However, results of some studies showed that the correlation between a high serum UA level and LVH was controversial (26, 27), indicating that there may be other factors, influencing the two parameters. Hence, a high serum UA level may induce LVH, rather than in all the patients.

The human apolipoprotein E gene, which has been widely studied in CVD, has three major isomers, E2, E3, and E4. Apolipoprotein plays a key role in cholesterol and triglyceride metabolism. Apolipoprotein E is a major component of very LDL (VLDL), which helps remove excess cholesterol from the blood, and transports it to the liver for metabolism. ApoE also helps remove chylomicrons and VLDL from the blood and binds to liver receptors, including LDL receptors, LDL receptor-associated proteins, and VLDL receptors as a ligand. The affinity of ApoE2 and hepatic lipoprotein receptor is poor, resulting in a reduction in the metabolism of lipoprotein decomposition of the triglycerides. Partial removal of TG from chylomicrons or VLDL creates remnants, and the ApoE4 is associated with the increased removal of these lipoproteins, leading to the high levels of cholesterol and LDL (28). It increases the risk of LVH as a risk factor for CVD (29), and the lipid levels of ε2 and ε3 carriers are significantly lower than those of ε4 carriers. Previous studies have shown that ApoE gene polymorphism is correlated with dyslipidemia and hyperuricemia. For instance, Moriwaki et al. (30) reported that the frequency of ApoE4 in patients with gout and hypertriglyceridemia is higher than that in patients with hypertriglyceridemia alone. In addition, ApoE4 is not only associated with obesity and alcoholism, but also can increase the incidence of hypertriglyceridemia in patients with gout as a risk factor for atherosclerosis. However, there is no study on the direct and indirect correlations between hyperuricemia and LVH. The results of our research showed a decreased risk of LVH in ε4 CHD patients with higher serum uric acid levels, while an increased risk in non-ε 4 carriers was recorded. To explain the association between serum UA level and LVH in patients with ε4 CHD, we assessed several hypotheses related to the effect of elevated serum UA levels on ε4 CHD patients. *In vitro* and *in vivo* studies have shown that UA is an important antioxidant in plasma, accounting for about 60% of the total antioxidant capacity of human plasma, and it can neutralize several harmful pro-oxidants, such as peroxynitrite, hydroxyl, and iron-containing free radicals, eliminating the activated erythrocyte-mediated organ damage by generating free radicals (31). Superoxide dismutase (SOD) is an effective antioxidant that can prevent the destruction of ROS, while UA can maintain the structure and function of extracellular SOD by preventing the oxidative neutralization mediated by atherosclerosis (32, 33). Data from a variety of populations, including healthy subjects, smokers, patients with type 1 diabetes, and patients with CVD, suggested that a high UA level may be a compensatory mechanism for significantly improving total antioxidant capacity and preserving endothelial function (34, 35). Patients with ApoE4 phenotype are mainly accompanied with hypercholesterolemia. An elevated serum UA level partially offsets the oxidative damage to vascular endothelium caused by hyperlipidemia through antioxidant effect, which may reduce the risk of atherosclerosis progressing to LVH to some extent. Although a growing number of evidence highlighted the antioxidant properties of UA in circulation, once inside the cell, UA promotes the oxidation of lipids, proteins, DNA, and carbohydrates. The association of hyperuricemia with metabolic abnormalities and the risk of CVD has been shown to be correlated with the increased pro-inflammatory activity of macrophages (36). CD11c, a β-2 integrin, is highly expressed in monocytes and macrophages, and it has a significant pro-inflammatory function, while CD206, also known as mannose receptor, is a c-type lectin, and it is mainly expressed in mouse and human macrophages and has anti-inflammatory effects (37–39). It has been previously reported that uricase inhibition of UA synthesis can reduce the number of CD11c+ monocytes in mice (40), suggesting an association between UA level and CD11c production. It has also been reported that CD206 expression of macrophages in synovial fluid of patients with gout tends to decrease compared with macrophages in patients with rheumatoid arthritis (41). In conclusion, macrophages have a pro-inflammatory function and may lose their anti-inflammatory ability in the case of elevated UA level. Studies have shown that UA stimulates the production of tumor necrosis factor-α (TNF-α), one of the pro-inflammatory cytokines, depending on the Toll-like receptor 4 (TLR4) signaling pathway. TLR4 is a transmembrane protein capable of recognizing multiple damage-related molecular patterns (DAMPs) and pathogen-related molecular patterns (PAMPs), including free fatty acids and lipopolysaccharide (LPS) (42, 43). Activation of TLR4 can induce the downstream activation of nuclear factor-kappa B (NF-KB), ultimately leading to the production of TNF-α (44). Therefore, we speculated that UA can induce the production of TNF-α through TLR4 activation. Under the UA action, the production of TNF-α, TLR4, and CD11c presents a typical dose-response relationship, and a plateau occurs when the maximum effect is exceeded and a saturation effect can be observed (45). Dose-response relationships attributed to interactions between ligands and their receptors suggest that they may be involved in a molecule capable of transporting uric acid within macrophages, such as URAT1, a transmembrane protein reported only in endothelial cells, adipocytes, and chondrocytes (46). The expression level of URAT1 in macrophages decreased with the increase of UA level, which may partly explain the saturation point of TNF-α, TLR4, and CD11c, leading to a decrease in their protein levels. Although URAT1 is not the only urate transporter, we speculate that the possible mechanism of the pro-inflammatory effect of uric acid on human macrophages may be associated with the dose-dependent response mode of URAT1. Compared with other genotypes, patients with ε4 had higher serum UA levels, and we hypothesized that the mechanism may be that the possible effect exceeded the dose-related maximum of URAT1 and NF-KB to reach a plateau, thus, there was no increase in LVH as serum UA levels continued to rise. For non-ε4 carriers, Cardona et al. (47) conducted a study on the correlation between ApoE allele and urate renal excretion in 68 patients with gout and 50 healthy controls, and found that the levels of TG and VLDL were significantly escalated in ApoE2 patients, while renal urate excretion was reduced that could be mediated by high levels of VLDL and ApoE2. Excretion rate of urate in patients with hyperuricemia is negatively correlated with plasma VLDL levels, further aggravating hyperuricemia. It was observed that hyperuricemia significantly increased the risk of LVH in patients without ε4 CHD in this study. The trend of influence of serum UA level on LVH is consistent with previously reported results.

The main limitation of this study is the cross-sectional design, which may affect the confirmation of causality, and further prospective study needs to be conducted to determine the relationship between serum UA level, ApoE polymorphism, and LVH. UA can increase the phagocytotic activity of macrophages by increasing the production of TNF-α, TLR4, and CD11c, and reduce the expression levels of CD206, CX3CR1, and CCR2. As a metabolic ligand, its pro-inflammatory effect on human macrophages partly depends on URAT1, which needs to be further studied.

In conclusion, a high serum UA level reduces the risk of LVH in ApoEε4 genotype, while increases the risk of LVH in non-ε4 genotype in CHD patients.

## Data availability statement

The original contributions presented in the study are included in the article/supplementary material, further inquiries can be directed to the corresponding author/s.

## Ethics statement

The studies involving human participants were reviewed and approved by the Clinical Research Ethic Committee of Beijing Chaoyang Hospital (Beijing, China). The patients/participants provided their written informed consent to participate in this study. Written informed consent was obtained from the individual(s) for the publication of any potentially identifiable images or data included in this article.

## Author contributions

YG designed the study. XZ and JL performed the research and wrote the manuscript. M-LZ and KL analyzed the data. MC participated in data collection. All the authors contributed to editorial changes in the manuscript and read and approved the final manuscript. YG also participated in the writing of the article.

## References

[1] Konstam MA Kramer DG Patel AR Maron MS Udelson JE Udelson. Left ventricular remodeling in heart failure current concepts in clinical significance and assessment. JACC Cardiovasc Imag. (2011) 4:98–108. 10.1016/j.jcmg.2010.10.008 21232712

[2] Heusch G Libby P Gersh B Yellon D Böhm M Lopaschuk G. Cardiovascular remodelling in coronary artery disease and heart failure. Lancet. (2014) 383:1933–43. 10.1016/S0140-6736(14)60107-0 24831770 PMC4330973

[3] Soliman EZ Ambrosius WT Cushman WC Zhang ZM Bates JT Neyra JA. Effect of intensive blood pressure lowering on left ventricular hypertrophy in patients with hypertension. SPRINT (systolic blood pressure intervention trial). Circulation. (2017) 136:440–50. 10.1161/CIRCULATIONAHA.117.028441 28512184 PMC5538944

[4] Gardin JM McClelland R Kitzman D Lima JA Bommer W Klopfenstein HS. M-mode echocardiographic predictors of 5- to 7-year incidence of coronary heart disease, stroke, congestive heart failure, and mortality in an elderly cohort (the cardiovascular health study). Am J Cardiol. (2001) 87:1051–7. 10.1016/S0002-9149(01)01460-6 11348601

[5] Lazzeroni D Rimoldi O Camici PG. From left ventricular hypertrophy to dysfunction and failure. Circ J. (2016) 80:555–64. 10.1253/circj.CJ-16-0062 26853555

[6] Yilmaz A Sechtem U. Diagnostic approach and differential diagnosis in patients with hypertrophied left ventricles. Heart. (2014) 100:662–71. 10.1136/heartjnl-2011-301528 23633547

[7] Katholi RE Couri DM. Left ventricular hypertrophy. Major risk factor in patients with hypertension update and practical clinical applications. Int J Hypertens. (2011) 2011:495349. 10.4061/2011/495349 21755036 PMC3132610

[8] Yoshimura A Adachi H Hirai Y Enomoto M Fukami A Kumagai E. Serum uric acid is associated with the left ventricular mass index in males of a general population. Int Heart J. (2014) 55:65–70. 10.1536/Ihj.13-170 24463929

[9] Buono F Spinelli L Giallauria F di Panzillo EA Di Marino S Ferrara F. Usefulness of satisfactory control of low-density lipoprotein cholesterol to predict left ventricular remodeling after a first ST-elevation myocardial infarction successfully re-perfused. Am J Cardiol. (2011) 107:1772–8. 10.1016/j.amjcard.2011.01.066 21529724

[10] Catena C Colussi G Capobianco F Brosolo G Sechi LA. Uricaemia and left ventricular mass in hypertensive patients. Eur J Clin Invest. (2014) 44:972–81. 10.1111/eci.12331 25186106

[11] Zeng C Cheng D Sheng X Jian G Fan Y Chen Y. Increased serum uric acid level is a risk factor for left ventricular hypertrophy but not independent of eGFR in patients with type 2 diabetic kidney disease. J Diabetes Res. (2017) 2017:5016093. 10.1155/2017/5016093 28713836 PMC5496120

[12] Eichner JE Dunn ST Perveen G Thompson DM Stewart KE Stroehla BC. Apolipoprotein E polymorphism and cardiovascular disease. A HuGE review. Am J Epidemiol. (2002) 155:487–95. 10.1093/aje/155.6.487 11882522

[13] Ward H Mitrou PN Bowman R Luben R Wareham NJ Khaw KT. APOE genotype, lipids, and coronary heart disease risk a prospective population study. Arch Int Med. (2009) 169:1424–9. 10.1001/archinternmed.2009.234 19667307

[14] Smith C Graham DI Murray LS Stewart J Nicoll JA. Association of APOE e4 and cerebrovascular pathology in traumatic brain injury. Journal Of Neurology Neurosurgery And Psychiatry. (2006) 77:363–6. 10.1136/jnnp.2005.074617 16484645 PMC2077683

[15] Trumble BC Stieglitz J Blackwell AD Allayee H Beheim B Finch CE. Apolipoprotein E4 is associated with improved cognitive function in Amazonian forager-horticulturalists with a high parasite burden. Faseb Journal. (2017) 31:1508–15. 10.1096/fj.201601084R 28031319 PMC5349792

[16] Sun YP Zhang B Miao L Wang XM Yu JH Luo L. Association of apolipoprotein E (ApoE) polymorphisms with risk of primary hyperuricemia in Uygur men, Xinjiang, China. Lipids Health Dis. (2015) 14:25. 10.1186/s12944-015-0025-2 25890021 PMC4446952

[17] Wu J Qiu L Guo XZ Xu T Cheng XQ Zhang L. Apolipoprotein E gene polymorphisms are associated with primary hyperuricemia in a Chinese population. PLos ONE. (2014) 9:e110864 10.1371/journal.pone.0110864 25356596 PMC4214707

[18] Ingelsson M Shin Y Irizarry MC Hyman BT Lilius L Forsell C. Genotyping of apolipoprotein E. comparative evaluation of different protocols. Curr Protoc Hum Genet. (2003) 9:14. 10.1002/0471142905.hg0914s38 18428347

[19] American Diabetes. Diagnosis and classification of diabetes mellitus. Diabetes Care. (2014) 37(Suppl 1):S81–90. 10.2337/dc14-S081 24357215

[20] Stone NJ Robinson JG Lichtenstein AH Bairey Merz CN Blum CB Eckel RH. American college of cardiology/American heart association task force on practice. 2013 ACC/AHA guideline on the treatment of blood cholesterol to reduce atherosclerotic cardiovascular risk in adults. a report of the American college of cardiology/American heart association task force on practice guidelines. J Am Coll Cardiol. (2014) 63(25 Pt B) 2889–934. 10.1016/j.jacc.2013.11.002 24239923

[21] Reiner Ž Catapano AL De Backer G Graham I Taskinen MR Wiklund O. Guidelines and Committees. ESC/EAS Guidelines for the management of dyslipidaemias. the Task Force for the management of dyslipidaemias of the European society of cardiology (ESC) and the European atherosclerosis society (EAS). Eur Heart J. (2011) 32:1769–818. 10.1093/eurheartj/ehr158 21712404

[22] Mancia G De Backer G Dominiczak A Cifkova R Fagard R Germano G. Management of arterial hypertension of the European society of and C. European society of. 2007 guidelines for the management of arterial hypertension. The task force for the management of arterial hypertension of the European society of hypertension (ESH) and of the European society of cardiology (ESC). J Hypertens. (2007) 25:1105–87 10.1097/HJH.0b013e3281fc975a 17563527

[23] Atherton JJ Bauersachs J UK AJ Carerj S Ceconi C Coca A. 2016 ESC guidelines for the diagnosis and treatment of acute and chronic heart failure. Eur J Heart Fail. (2016) 18:891–975. 10.1002/ejhf.592 27207191

[24] Johnson RJ Kang DH Feig D Kivlighn S Kanellis J Watanabe S. Is there a pathogenetic role for uric acid in hypertension and cardiovascular and renal disease? Hypertension. (2003) 41:1183–90. 10.1161/01.HYP.0000069700.62727.C5 12707287

[25] Onat A Uyarel H Hergenc G Karabulut A Albayrak S Sari I. Serum uric acid is a determinant of metabolic syndrome in a population-based study. Am J Hypertens. (2006) 19:1055–62. 10.1016/j.amjhyper.2006.02.014 17027827

[26] G Mule G Nardi E Costanzo M Mogavero M Guarino L Viola T. Absence of an independent association between serum uric acid and left ventricular mass in Caucasian hypertensive women and men. Nutri Metabol Cardiovasc Dis. (2013) 23:715–22. 10.1016/j.numecd.2012.01.007 22494808

[27] Cuspidi C Valerio C Sala C Meani S Esposito A Zanchetti A. Lack of association between serum uric acid and organ damage in a never treated essential hypertensive population at low prevalence of hyperuricemia. J Hypertens. (2007) 25:S97–8. 10.1016/j.amjhyper.2007.01.013 17531928

[28] Elzen PV Garg S León L Brigl M Leadbetter EA Gumperz JE. Apolipoprotein-mediated pathways of lipid antigen presentation. Nature. (2005) 437:906–10. 10.1038/nature04001 16208376

[29] Kand'ár R Žáková P MuŽáková V. Monitoring of antioxidant properties of uric acid in humans for a consideration measuring of levels of allantoin in plasma by liquid chromatography. Clin Chim Acta. (2006) 365:49–56. 10.1016/j.cca.2005.09.002 16194528

[30] Moriwaki Y Yamamoto T Takahashi S Tsutsumi Z Higashino K. Apolipoprotein E phenotypes in patients with gout. Relation with hypertriglyceridaemia. Ann Rheum Dis. (1995) 54:351–4. 10.1136/ard.54.5.351 7794039 PMC1005593

[31] Becker BF. Towards the physiological function of uric acid. Free Radic Biol Med. (1993) 14:615–31. 10.1016/0891-5849(93)90143-I 8325534

[32] Hink HU Fukai T. Extracellular superoxide dismutase, uric acid, and atherosclerosis. Cold Spring Harb Symp Quant Biol. (2002) 67:483–90. 10.1101/sqb.2002.67.483 12858574

[33] HHink HU Santanam N Dikalov S McCann L Nguyen AD Parthasarathy S. Peroxidase properties of extracellular superoxide dismutase. Role of uric acid in modulating in vivo activity. Arterioscler Thromb Vasc Biol. (2002) 22:1402–8. 10.1161/01.ATV.0000027524.86752.02 12231557

[34] Waring WS Convery A Mishra V Shenkin A Webb DJ Maxwell SR. Uric acid reduces exercise-induced oxidative stress in healthy adults. Clin Sci. (2003) 105:425–30. 10.1042/CS20030149 12801243

[35] Waring WS McKnight JA Webb DJ Maxwell SR. Uric acid restores endothelial function in patients with type 1 diabetes and regular smokers. Diabetes. (2006) 55:3127–32. 10.2337/db06-0283 17065352

[36] Haryono A Nugrahaningsih DA Sari DC Romi MM Arfian N. Reduction of serum uric acid associated with attenuation of renal injury, inflammation and macrophages M1/M2 ratio in hyperuricemic mice model. Kobe J Med Sci. (2018) 64:E107–14. 30666040 PMC6347045

[37] Arnold IC Mathisen S Schulthess J Danne C Hegazy AN Powrie F. CD11c(+) monocyte/macrophages promote chronic Helicobacter hepaticus-induced intestinal inflammation through the production of IL-23. Mucosal Immunol. (2016) 9:352–63. 10.1038/mi.2015.65 26242598 PMC4650208

[38] Torres-Castro I Arroyo-Camarena ÚD Martínez-Reyes CP Gómez-Arauz AY Dueñas-Andrade Y Hernández-Ruiz J. Human monocytes and macrophages undergo M1-type inflammatory polarization in response to high levels of glucose. Immunol Lett. (2016) 176:81–9. 10.1016/j.imlet.2016.06.001 27269375

[39] Nawaz A Aminuddin A Kado T Takikawa A Yamamoto S Tsuneyama K. CD206(+) M2-like macrophages regulate systemic glucose metabolism by inhibiting proliferation of adipocyte progenitors. Nat Commun. (2017) 8:286. 10.1038/s41467-017-00231-1 28819169 PMC5561263

[40] Kool M Soullié T Van Nimwegen M Willart MA Muskens F Jung S. Alum adjuvant boosts adaptive immunity by inducing uric acid and activating inflammatory dendritic cells. J Exp Med. (2008) 205:869–82. 10.1084/jem.20071087 18362170 PMC2292225

[41] Jeong JH Jung JH Lee JS Oh JS Kim YG Lee CK. Prominent inflammatory features of monocytes/macrophages in acute calcium pyrophosphate crystal arthritis. A comparison with acute gouty arthritis. Immune Netw. (2019) 19:e21. 10.4110/in.2019.19.e21 31281718 PMC6597439

[42] Rocha DM Caldas AP Oliveira LL Bressan J Hermsdorff HH. Saturated fatty acids trigger TLR4-mediated inflammatory response. Atherosclerosis. (2016) 244:211–5. 10.1016/j.atherosclerosis.2015.11.015 26687466

[43] Park BS Lee JO. Recognition of lipopolysaccharide pattern by TLR4 complexes. Exp Mol Med. (2013) 45:e66. 10.1038/emm.2013.97 24310172 PMC3880462

[44] Harada K Ohira S Isse K Ozaki S Zen Y Sato Y. Lipopolysaccharide activates nuclear factor-kappaB through toll-like receptors and related molecules in cultured biliary epithelial cells. Lab Invest. (2003) 83:1657–67. 10.1097/01.LAB.0000097190.56734.FE 14615419

[45] Salahudeen MS Nishtala PS. An overview of pharmacodynamic modelling, ligand-binding approach and its application in clinical practice. Saudi Pharm J. (2017) 25:165–75. 10.1016/j.jsps.2016.07.002 28344466 PMC5355565

[46] Zhang B Duan M Long B Zhang B Wang D Zhang Y. Urate transport capacity of glucose transporter 9 and urate transporter 1 in cartilage chondrocytes. Mol Med Rep. (2019) 20:1645–54. 10.3892/mmr.2019.10426 31257523 PMC6625399

[47] Cardona F Tinahones FJ Collantes E Garcia-Fuentes E Escudero A Soriguer F. Response to a urate-lowering diet according to polymorphisms in the apolipoprotein AI-CIII-AIV cluster. J Rheumatol. (2005) 32:903–5. 10.1016/j.jbspin.2004.06.007 15868628

